# Inscuteable Regulates the Pins-Mud Spindle Orientation Pathway

**DOI:** 10.1371/journal.pone.0029611

**Published:** 2012-01-10

**Authors:** Jonathon F. Mauser, Kenneth E. Prehoda

**Affiliations:** Institute of Molecular Biology and Department of Chemistry, University of Oregon, Eugene, Oregon, United States of America; Institute for Research in Biomedicine, Spain

## Abstract

During asymmetric cell division, alignment of the mitotic spindle with the cell polarity axis ensures that the cleavage furrow separates fate determinants into distinct daughter cells. The protein Inscuteable (Insc) is thought to link cell polarity and spindle positioning in diverse systems by binding the polarity protein Bazooka (Baz; aka Par-3) and the spindle orienting protein Partner of Inscuteable (Pins; mPins or LGN in mammals). Here we investigate the mechanism of spindle orientation by the Insc-Pins complex. Previously, we defined two Pins spindle orientation pathways: a complex with Mushroom body defect (Mud; NuMA in mammals) is required for full activity, whereas binding to Discs large (Dlg) is sufficient for partial activity. In the current study, we have examined the role of Inscuteable in mediating downstream Pins-mediated spindle orientation pathways. We find that the Insc-Pins complex requires Gαi for partial activity and that the complex specifically recruits Dlg but not Mud. *In vitro* competition experiments revealed that Insc and Mud compete for binding to the Pins TPR motifs, while Dlg can form a ternary complex with Insc-Pins. Our results suggest that Insc does not passively couple polarity and spindle orientation but preferentially inhibits the Mud pathway, while allowing the Dlg pathway to remain active. Insc-regulated complex assembly may ensure that the spindle is attached to the cortex (via Dlg) before activation of spindle pulling forces by Dynein/Dynactin (via Mud).

## Introduction

Precise positioning of the mitotic spindle is critical for a broad range of processes, including cell type differentiation and tissue organization [1], [2]. For example, in the asymmetric division of Drosophila neuroblasts proper segregation of fate determinants requires that the spindle align with the axis of apical/basal cell polarity [3], [4]. Incorrect spindle orientation has been implicated in a number of pathologies, including tumorigenesis [5].

During the neuroblast asymmetric division, cell fate determinants become polarized by metaphase. Factors important for differentiation of the basal daughter cell localize to the basal cell cortex, whereas factors that maintain neuroblast identity localize to the apical cortex [5]. During cytokinesis, the two polarity domains become separated by the cleavage furrow such that the apical daughter cell retains the neuroblast identity and the basal cell differentiates into a neuron or glial cell. The mitotic spindle plays a crucial role in specifying the position of the cleavage furrow [6]–[8] and thus proper fate determinant segregation requires alignment of the spindle with the polarity axis.

**Figure 1:**
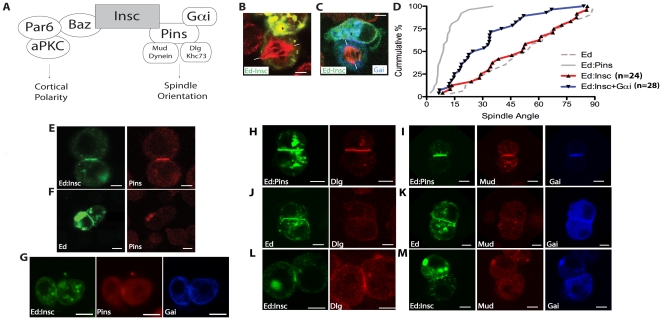
Inscuteable-mediated orientation of the mitotic spindle requires Gαi. a, current model of Inscuteable function. Insc serves as a link between the apical PAR complex and the spindle-orienting Pins-Gái complex. b, Ed-Insc (green) transfected S2 cells randomly orient the mitotic spindle (red) with respect to the region that is enriched in Ed. Spindle alignment is measured by drawing a vector from the center of the crescent (arrow) to the center of the mitotic spindle and then along the axis (dashes). c, Expression of Gái (blue) with Ed-Insc (green)is able to confer moderate spindle orienting activity. d, Cumulative percentage plot of spindle angles measured in the S2 Echinoid induced-polarity assay for Ed-Insc and Ed-Insc+Gái compared to previously-published data [17]. In these plots, the cumulative percentage of cells with a spindle angle below a particular value (x-axis) is shown. High spindle orienting activity corresponds to a deflection to lower spindle angles whereas no activity is a line across the diagonal. e, Ed-Insc expression in S2 cells is sufficient to robustly recruit endogenous Pins from the cytoplasm to the region of Ed enrichment. f, Ed alone is unable to polarize endogenous Pins. e, g, Ed-Insc induces colocalization of endogenous Pins with overexpressed Gai. h, Ed-Pins is able to recruit endogenous Dlg. i, Co-expression of Gαi with Ed-Pins results in robust recruitment of endogenous Mud. j,k Ed-GFP is unable to recruit endogenous Dlg or Mud to the induced-polarity cortical domains. l, Ed-Insc is able to recruit Dlg to the cortex, similar to cells expressing Ed-GFP-Pins. m, Ed-Insc is not able to recruit Mud (red) to the Ed-crescent, even in the presence of Gái. Scale bars for all panels represent 5 µm.

Coupling of polarity and spindle orientation is thought to be mediated by the protein Inscuteable (Insc) because of its ability to bind components from both systems (Fig. 1A) [3]. The functional region of Inscuteable, the central Ankyrin-repeat-like domain, has been previously characterized [9]. Insc interacts with Bazooka (Baz; aka Par-3), a component of the apical Par polarity complex that also includes the proteins Par-6, and atypical Protein Kinase C (aPKC) [3], [9], [10]. Insc also binds Partner of Inscuteable (Pins), which regulates neuroblast spindle orientation [11], [12]. In *insc* mutant neuroblasts, both cell polarity and spindle orientation are defective [13], [14].

Insc is thought to act as a localization signal for Pins. Pins, in turn, activates two downstream pathways that participate in mitotic spindle positioning. The Pins tetratricopeptide repeats (TPR) motifs bind Mushroom body defect (Mud; NuMA in mammals) [15]. In mammals, NuMA (Nuclear Mitotic Apparatus), the Mud ortholog, has been previously been shown to be abundant in the nuclei of interphase cells and to play an essential role during mitotic spindle assembly and alignment during mitosis. Mud/NuMA in turn are thought to recruit the Dynein/Dynactin complex, which can generate pulling forces on astral microtubules from its minus-end directed motor activity [16]. Pins also contains a central Linker domain that is phosphorylated by the mitotic kinase Aurora A. The phosphorylated Linker domain binds Discs large (Dlg, PSD-95 in mammals) which acts to recruit the plus-end directed kinesin Khc73 (Kinesin-3, GAKIN, Kif13B in mammals) [14], [17].

Whether Insc is a passive scaffold that simply provides a physical link between polarity and spindle position, or if Insc somehow actively regulates the two pathways downstream of Pins has been unclear. Here we examine the effect of Insc on Pins-mediated spindle orientation using an induced polarity cell culture system [17]. This system allows for precise control of the components that are placed on the cortex and can be subsequently interrogated for spindle orienting activity.

## Results

### Polarized Inscuteable recruits Pins but lacks spindle orientation activity

In the current model for Insc-based coupling of polarity and spindle orientation, Insc recruits Pins, which in turn recruits the downstream effectors Mud and Dlg (Fig. 1A) [11], [15], [17], [18]. In previous work, we found that Insc lacked activity in an induced polarity spindle orientation assay [17]. In this assay, proteins are fused to the cytoplasmic domain of the adhesion protein Echinoid (Ed) and transiently transfected into cultured Drosophila S2 cells. Cell clustering leads to polarization of the Ed fusion protein at sites of cell-cell contact and the angle of the spindle to the center of the induced crescent can be measured. Although we have observed that the spindle aligns with polarized Ed-PinsTPR-LINKER fusions, the spindle is randomly oriented in cells with polarized Ed-Insc fusions (Fig. 1B, D).

To investigate why Ed-PinsTPR-LINKER orients the spindle but Ed-Insc fails to do so, we first determined if Pins is recruited to Ed-Insc. Endogenous Pins protein strongly colocalizes with Ed-Insc (Fig. 1E). However in cells with polarized Ed alone Pins remains in the cytoplasm (Fig. 1F). Thus, we conclude that Ed-Insc recruits Pins, yet is unable to orient the spindle. Pins is known to be autoinhibited for Mud-binding by an intramolecular interaction between its NH2- and COOH termini [19], [20]. This autoinhibition is relieved by binding of the heterotrimeric G-protein subunit, Gαi. Ed-Insc may not exhibit spindle orientation activity because the Pins that it recruits has not been activated. We expressed Gαi with Ed-Insc to ensure that Insc-bound Pins is activated. Ed-Insc and Gαi co-expression leads to the formation of an Insc-Pins-Gαi complex at the crescent, but only a moderate amount of spindle orienting activity, similar to cells with polarized Pins in which only the downstream Dlg pathway, but not the Mud pathway, has been activated [17] (Fig. 1C, D, G).

### Insc-Pins recruits Dlg but not Mud

The Insc-Pins-Gαi complex may not fully orient the mitotic spindle because of failure to recruit downstream effectors that are normally brought to the cortex by Pins. We tested for recruitment of the two known Pins spindle orientation pathways, Dlg and Mud. In cells expressing Ed-Pins, Dlg is robustly recruited to the cell-cell contacts (Fig. 1H) and co-expression of Ed-Pins with Gαi results in strong Mud recruitment (Fig. 1I). Dlg and Mud recruitment is specific as it is not observed in cells expressing Ed-GFP (Fig. 1 J, K).

We next examined whether Dlg and Mud are recruited to Ed-Insc. Dlg is recruited to Ed-Insc in a similar manner as Ed-Pins (Fig. 1L). However, while Pins-Gαi can recruit Mud, Insc-Pins-Gαi is unable to do so (Fig. 1M). Thus, Insc appears to regulate Pins complex assembly, leading to preferential activation of only one of the two spindle orientation pathways, with the effect of an overall reduction in spindle orientation activity.

### Insc represses Pins-mediated spindle orientation

**Figure 2:**
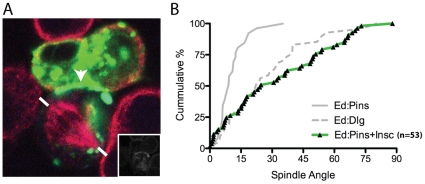
Expression of Inscuteable in cells expressing constitutively-active Pins reduces spindle orientation to Dlg-like levels. a, Co-expression of Ed-Pins 1–466 (green), which robustly orients the mitotic spindle, with Inscuteable (inset), reduces the levels of spindle orientation in adherent, polarized S2 cells. b, Cumulative percentage plot of spindle angles measured in cells co-expressing Ed-Pins 1–466 and Inscuteable compared to published data [17].

Ed-Insc cannot fully orient the spindle even though it recruits Pins and Gαi. Polarized Ed-Pins coexpressed with Gαi, however, has full spindle-orienting activity [17]. These data suggest that Insc preferentially inhibits Pins spindle orienting activity. To further investigate if Insc inhibits Pins-mediated spindle orientation, we expressed Insc in cells with polarized Ed-PinsTPR-LINKER, a construct lacking autoinhibition that, when expressed on its own, fully aligns the mitotic spindle (Fig. 2A) [17]. We observed that Insc is recruited to Ed-PinsTPR-LINKER crescents (Fig. 2A, inset) and that the presence of Insc reduces its spindle orienting activity to a level indistinguishable from the Dlg pathway alone (Fig. 2B). Thus, we conclude that Insc inhibits the spindle-orienting activity of PinsTPR-LINKER.

### The Pins TPR domains bind Inscuteable

Why might Insc-Pins recruit Dlg, but not Mud? One possible explanation is that Insc and Mud compete for binding to Pins. Mud is known to bind the Pins tetratricopeptide repeats (TPRs) [15], [19], [20]. To identify the Pins region responsible for binding Insc, we performed a deletion analysis using affinity pulldowns with purified proteins. Pins contains seven TPR repeats followed by a flexible Linker domain and three GoLoco motifs that bind Gαi. GST-fusions of full-length Pins, the GoLoco region, and the TPR region were generated and incubated with a purified MBP-fusion of the central Ankyrin-repeat containing domain of Inscuteable (MBP-Insc) [9].

**Figure 3:**
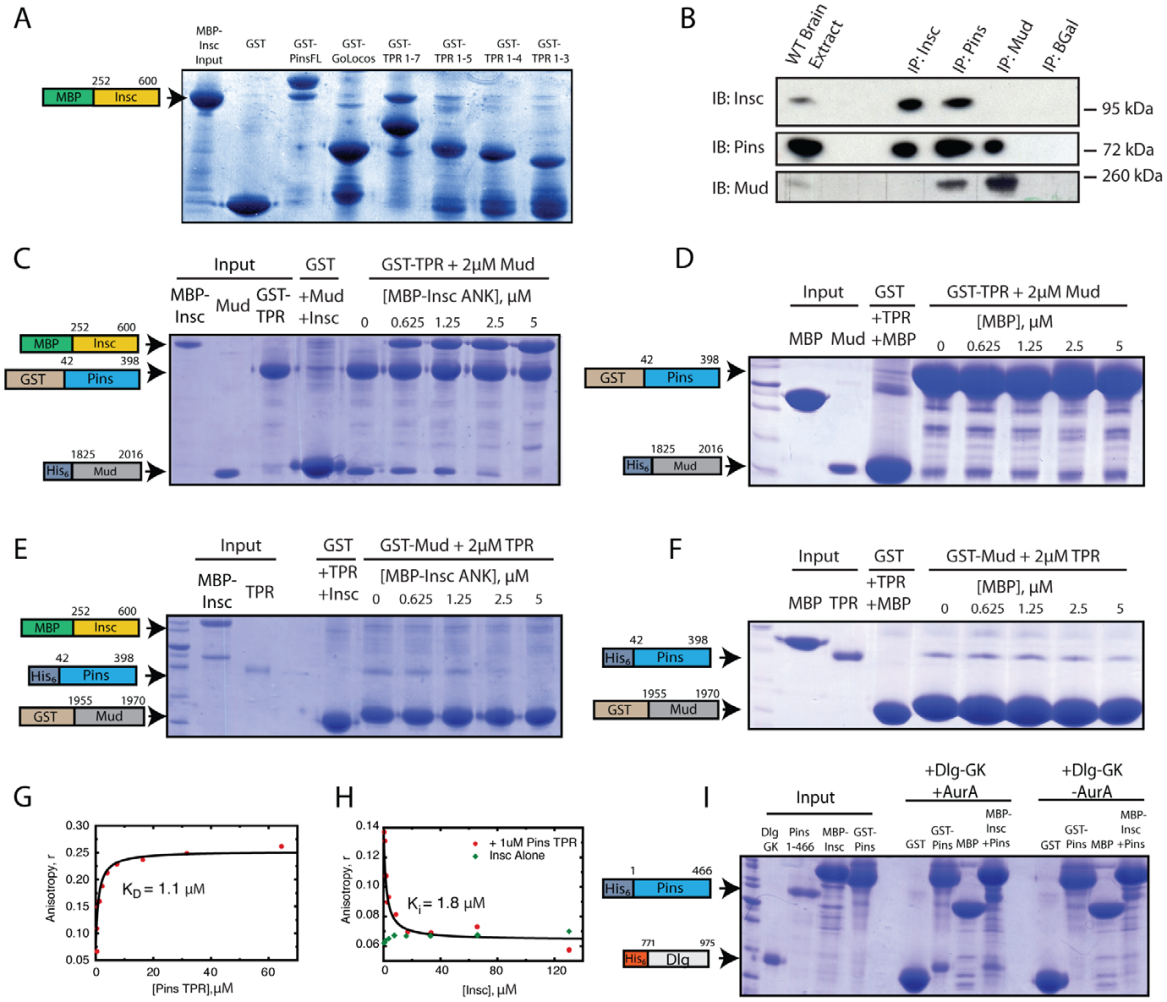
Inscuteable competes with Mud, but not Dlg, for binding to Pins. a, GST-pulldowns of Inscuteable with different Pins constructs reveals that Inscuteable binds specifically to constructs containing the full array of Pins TPRs. b, Coimmunoprecipitations of endogenous proteins from wild-type L3 brain extracts demonstrate that Inscuteable and Mud form exclusive complexes with Pins. c, GST-pulldown using GST-Pins TPRs incubated with a constant amount of Mud and increasing MBP-Insc reveals effective competition between Mud & Insc for binding to Pins. d, A control titrations of MBP alone do not result in dissociation of Mud from GST-Pins. e, GST-pulldown using GST-Mud incubated with constant 2 uM Pins TPR and increasing amounts of MBP-Insc results in an approximately 1∶1 stoichiometric dissociation of Pins TPRs from GST-Mud. f, A control titration of MBP alone does not result in disruption of Pins-Mud binding. g, Fluorescence anisotropy of TMR-Mud with increasing amounts of Pins TPRs exhibits a robust association profile. h, Addition of Inscuteable to a pre-formed complex of 100 nM TMR-Mud & 1 uM Pins causes a dissociation of the Mud-Pins complex & reduction of TMR-Mud anisotropy. i, GST-pulldown using *in vitro* Aurora-A phosphorylated Pins 1–466 results in complex formation with the Dlg GK domain. AurA treatment of a pre-formed MBP-Insc/Pins 1–466 complex likewise is able to form a complex with the Dlg GK domain.

All constructs containing the full set of 7 Pins TPRs are able to bind Insc, whereas those lacking these repeats, such as the COOH-terminal GoLoco domains, are unable to bind Insc (Fig. 3A). Further TPR truncations were also performed to find the minimal TPR region required for binding to Insc. While binding of Insc to Pins is detectable using a constructs consisting of the full set of TPRs as well as TPRs 1–5, all seven TPRs are required for high-affinity association with Insc (Fig. 3A). Since Mud/NuMA have also been shown to require a full array of TPRs for high-affinity binding [15] both Insc and Mud bind to the Pins TPR motifs.

### Mud and Insc compete for binding to Pins

As Insc and Mud both bind the Pins TPRs, we examined whether Insc and Mud could bind simultaneously to Pins. Extracts were prepared from the brains of wild-type third-instar larvae and complex formation was examined by immunoprecipitation of the endogenous components. As expected, we observed both Insc and Mud in Pins immunoprecipitates. However, in Insc immunoprecipitates, we observed Pins but Mud was not present (Fig. 3B). The lack of Mud in Insc immunoprecipitates suggested that Pins forms mutually exclusive complexes with Insc and Mud. Likewise Insc was also not observed in Mud immunoprecipitates (Fig. 3B). Thus, Insc and Mud appear to form mutually exclusive complexes with Pins.

We further tested for competition between Insc and Mud for Pins using qualitative pull-downs with purified proteins. The Mud-Pins complex can be readily formed on glutathione agarose, using GST-PinsTPR and a purified Mud fragment containing the minimal TPR binding domain (Fig. 3C) [15]. Introduction of MBP-Insc to these reactions dissociates Pins from Mud, resulting in switching to the Pins-Insc complex, a result consistent with competition between Insc and Mud for Pins. This effect is not observed with identical concentrations of MBP alone (Fig. 3D). GST- pulldowns with pre-formed complexes of GST-Mud and Pins TPR were likewise disrupted by addition of MBP-Insc (Fig. 3E). This effect is not observed when MBP alone is titrated into identical reactions (Fig. 3F).

Finally, we examined Insc and Mud competition using fluorescence anisotropy. We labeled a peptide representing the minimal region of Mud that binds Pins with the fluorophore tetramethylrhodamine (TMR-Mud). Binding of Pins causes a significant increase in TMR-Mud anisotropy due to complex assembly (Fig. 3G). Insc addition to a pre-formed complex of Pins & Mud leads to a decrease in TMR-Mud anisotropy to a value consistent with free peptide. No effect of Insc was observed when Pins is not present, indicating that TMR-Mud does not bind directly to Insc (Fig. 3H).

The decrease in anisotropy is a further indication that Insc competes for Mud binding and allows for calculation of the Insc affinity for Pins of K_d_ = 5 µM. Interestingly, this affinity is somewhat lower than the Pins-Mud interaction (Kd = 1.1 µM). Together, the immunoprecipitation, pull-down, and fluorescence anisotropy results indicate that Mud and Insc compete for Pins binding.

### Discs large, Inscuteable, and Pins form a stable ternary complex

Pins can also bind the downstream effector Dlg through its phosphorylated Linker domain. Activation of the Dlg pathway leads to partial spindle orienting activity, similar to that observed for the Insc-Pins complex. To determine if Insc-Pins can bind Dlg, we examined their binding in a pull-down experiment. Interaction of Pins with Dlg requires Aurora-A phosphorylation of the Pins Linker domain and we observed phosphorylation-dependent formation of an Insc-Pins-Dlg ternary complex (Fig. 3I). This result indicates that while Insc represses Mud binding to the Pins TPRs, it has no effect on regulating the downstream Dlg pathway.

## Discussion

Spindle positioning is important in many physiological contexts [21]–[23]. At a fundamental level, spindle orientation determines the placement of the resulting daughter cells in the developing tissue, which is important for correct morphogenesis and tissue organization [24]–[25]. In other contexts, such as asymmetric cell division, spindle position ensures proper segregation of fate determinants and subsequent differentiation of daughter cells. We have examined the function of a protein thought to provide a “passive” mark on the cortex for subsequent recruitment of the spindle orientation machinery. During neuroblast asymmetric cell division, Insc has been thought to mark the cortex based on the location of the Par polarity complex.

Ectopic expression of Insc in cells that normally do not express the protein has revealed that it is sufficient to induce cell divisions oriented perpendicular to the tissue layer, reminiscent of neuroblast divisions [13], [26], [27]. Expression of the mammalian ortholog of Inscuteable, mInsc, in epidermal progenitors has shown that this phenotype is not completely penetrant over time [27]. Expression of mInsc leads to a transient re-orientation of mitotic spindles, in which mInsc and NuMA initially co-localize at the apical cortex. After prolonged expression, however, the epidermal progenitors return to dividing along the tissue polarity axis, a scheme in which mInsc and NuMA no longer co-localize. These results indicate that Insc and Mud can be decoupled from one another.

We have examined the effect of Insc-Pins complex formation both in an induced polarity spindle orientation assay and in *in vitro* binding assays. Our results indicate that Insc plays a more active role in spindle positioning than previously appreciated. Rather than passively coupling polarity and spindle positioning systems, Insc acts to regulate the activity of downstream Pins pathways. We have shown that the Dlg pathway is unaffected by Inscuteable expression while the Mud pathway is inhibited by Insc binding.

Recent work on the mammalian versions of these proteins explains the structural mechanism for competition between the Insc-Pins and Pins-Mud complexes [28]. The binding sites on Pins for these two proteins overlap making binding mutually exclusive because of steric considerations. The observation of Insc dissociation of the Pins-Mud complex in Drosophila (this work) and mammalian proteins (LGN-NuMA) [28] suggests that Insc regulation of Mud-binding is a highly conserved behavior.

This competition between Mud and Insc for Pins binding is consistent with previous work done with a chimeric version of Inscuteable/Pins [29]. This protein, in which the Pins TPR domain was replaced with the Inscuteable Ankyrin-repeat domain, bypasses the Insc-Pins recruitment step of apical complex formation. In these cells, the chimeric Insc-Pins protein was able to rescue apical/basal polarity and spindle orientation in metaphase *pins* mutant neuroblasts. As this protein lacks the Mud-binding TPR domain, Mud binding to Pins is not absolutely necessary for spindle alignment. Importantly, the PinsLINKER domain is still intact in the Insc-Pins fusion, implying that Dlg, not Mud, function is sufficient for partial activity, as observed in the S2 system [17].

The Mud and Dlg pathways may play distinct roles in spindle positioning. The Dlg pathway, through the activity of the plus-end directed motor Khc73, may function to attach the cortex to the spindle through contacts with astral microtubules [14]. In contrast, the Mud pathway, through the minus-end directed Dynein/Dynactin generates force to draw the centrosome towards the center of the cortical crescent [16]. Fusion of the Pins TPR motifs, which recruit Mud, to Echinoid does not lead to spindle alignment, indicating that the Mud pathway is not sufficient for spindle alignment. The PinsLINKER domain does have partial activity on its own, however, and when placed in cis with the TPRs leads to full alignment [17]. In this framework, the function of Insc may be temporal control, ensuring that microtubule attachment by the Dlg pathway occurs before the force generation pathway is activated.

**Figure 4:**
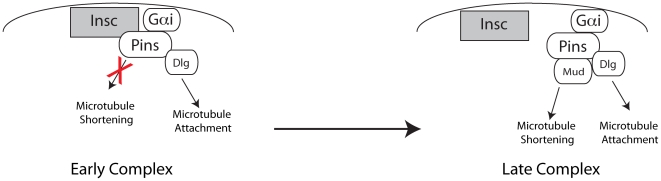
Proposed model for Inscuteable regulation of spindle orientation. a, In early interphase, Inscuteable recruits cortical Gαi-Pins to the apical cortex. Insc-bound Pins can scaffold for Dlg, allowing for early microtubule attachment, but inhibits binding of Mud, preventing ectopic microtubule shortening. b, after nuclear envelope breakdown and trafficking along the mitotic spindle, Mud from astral microtubules competes Pins away from Insc and allows for microtubule shortening.

In the temporal model of Insc function, what might cause the transition from the Insc-Pins-Dlg complex, which mediates astral microtubule attachment, to the Mud-Pins-Dlg complex, which generates spindle pulling forces? By early prophase, Inscuteable recruits Pins and Gαi to the apical cortex [14]. During this phase of the cell cycle, Mud is localized to the nucleus in high concentration [30], [31]. Apically-localized Pins binds Dlg, creating an apical target for astral microtubules (Fig. 4A). During early phases of mitosis, Inscuteable would serve to inhibit binding of low concentrations of cytoplasmic Mud to the Pins TPRs to prevent spurious activation of microtubule shortening pathways. After nuclear envelope breakdown, Mud enters the cytoplasm in greater concentrations [31] and could then act to compete with Insc for binding to Pins (Fig. 4B), allowing Pins output to be directed into microtubule-shortening pathways. Future work will be directed towards testing additional aspects of this model.

## Materials and Methods

### Molecular cloning, protein expression and purification

Constructs encoding Drosophila Pins, Inscuteable, and Mud have been described [9], [18]. Residues 252–600 of Inscuteable, including the central Ankyrin-repeat containing region, were used for all experiments. Residues 1–466 of Pins, corresponding to the TPR+LINKER domains, 42–398, corresponding to the TPR domain, and 372–658, corresponding to the three GoLoco domains were used for Inscuteable binding studies. Mud residues 1825–2016, which includes the minimal Pins-binding domain, were also amplified for binding assays.

Echinoid (Ed) fusion constructs were made in pMT-V5 (Invitrogen, Carlsbad, CA), replacing the Ed cytoplasmic domain with a visualization tag and the protein of interest at the COOH terminus (e.g., Ed-GFP-Insc). Proteins for pull down and anisotropy experiments were expressed in Escherichia coli strain BL21(DE3) using pGEX 4T-1-based vectors for GST fusions, pMAL-c2 based-vectors for MBP fusions, and pBH-based vectors for hexahistidine fusions. GST-fusion proteins were purified on glutathione-agarose resin and washed with a large excess of GST pulldown buffer (10 mM HEPES pH 7.5/100 mM NaCl/1 mM DTT). The resin was then used for subsequent GST-pulldowns. MBP-fusion proteins were purified on amylose resin (New England Biolabs), washed with three bed-volumes of PBS+1% Triton X-100 and one bed-volume of PBS. Proteins were eluted using PBS+1M Methyl-α-D-glucopyranoside (Sigma-Aldrich). Hexahistidine-fusion proteins were purified on Ni-NTA agarose resin (Qiagen). The incubated resin was then washed with a large excess of cell lysis buffer (50 mM NaPO4/150 mM NaCl/10 mM imidazole). Samples were then eluted with elution buffer (50 mM NaPO4/150 mM NaCl/300 mM imidazole).

### Transfection and S2 Cell Experiment

S2 cells were grown and cultured at room temperature in Schneider's Insect Media (Sigma) supplemented with 10% fetal bovine serum. Echinoid polarity assays were carried out as described previously [14]. In short, 1×10∧6 cells were transiently transfected with pMT-V5 fusion constructs (400 ng each) using Effectene (QIAGEN) reagent according to manufacturer protocol. 24–48 hrs after transfection, protein expression was induced by incubation with CuSO4 (500 µM) for 24 hr. Cells were harvested by centrifugation and the media was replaced. These cells were then shaken (175 RPM) for 2–3 hr to induce Ed-mediated cell-cell clusters. These cells were then were plated on glass coverslips and allowed to incubate for 3 hr to allow for cell divisions to occur.

### Immunostaining

For immunostaining, S2 cells were fixed for 20 min in 4% paraformaldehyde, stained, and imaged on a Leica SP2 confocal microscope with a 63×1.4 NA lens. Antibodies and dilutions were as follows: rabbit Gαi, 1∶1000 [17], mouse Dlg, 1∶250 (Developmental Studies Hybridoma Bank, Iowa); rabbit Mud 1∶1000 (gift from Y. Bellaiche); rat Pins, 1∶500 [10]; rat tubulin, 1∶1000 (Abcam); rabbit Insc 1∶1000 (gift from W.Chia), rabbit HA, 1∶1000 (Covance).

### Immunoprecipitation and Western Blots

For western blot lysate inputs, 20 µg of total protein from brain extracts were used per lane. Immunoprecipitation from larval brain extracts was carried out using antibodies bound to protein G sepharose (GE Healthcare) according to the manufacturer's instructions. 40 brains from L3 larvae were dissected and homogenized by douncing in 300 uL sample buffer (50 mM HEPES pH 7.5/150 mM NaCl/1 mM DTT/0.1% Triton X-100/EDTA-free Protease Inhibitors (Roche)). Extracts were then centrifuged twice for 10 minutes each at 10,000 rpm to pellet insoluble cell debris. The resulting supernatant was then precleared with protein G sepharose and incubated with antibody-bound resin. Following three washes in sample buffer, the resin was heated to 95°C in SDS loading buffer (1% SDS/100 mM DTT/50 mM Tris pH. 7.5/0.003% bromophenol blue). Immunoprecipitates were resolved on SDS-PAGE followed by western blotting.

### Measuring Cortical Polarity, Spindle Orientation, and Centrosome Alignment

Spindle alignment measurements were made as described previously [17]. Briefly, spindle angles were measured with a vector perpendicular to the center of the Ed crescent and a vector matching the spindle or connecting the spindle poles. The angle between these two vectors was then assessed.

### In Vitro Binding Assays

GST pull-down assays have been described [19]. Briefly, ligands were added to glutathione agarose with adsorbed GST fusion proteins in binding buffer (10 mM Hepes/100 mM NaCl/1 mM DTT) at the indicated concentrations to a final reaction volume of 50 µl and incubated at room temperature for 15 min before washing, elution, and analysis by gel electrophoresis.

Fluorescence anisotropy binding assays were as described [19]. A peptide containing the sequence of Mud residues 1955–1970 and an NH2-terminal cysteine was labeled with tetramethylrhodamine maleimide (Life Technologies) according to the manufacturer's instructions. The labeled protein was purified by reverse-phase HPLC. For binding experiments, solutions were prepared with increasing amount of ligand and constant dye-labeled component (100 nM) in binding buffer with the temperature maintained at 20°C by using a circulating water bath. Data series were fit to an equation describing 1∶1 binding.

### In Vitro Kinase Assays

Recombinant Aurora-A kinase was purchased from Millipore (Billerica, MA). Pins constructs (10 µg) and Aurora-A (100 ng) were diluted in ice-cold assay buffer (20 mM Tris [pH 7.4], 100 mM NaCl, 1 mM DTT, 10 mM MgCl2, and 10 µM ATP). These reactions were then moved to room temperature for 30 minutes. Reactions were then chilled on ice and added to affinity pulldown resin for pulldown experiments.

## References

[1] Cabernard C, Doe CQ (2009). Apical/basal spindle orientation is required for neuroblast homeostasis and neuronal differentiation in Drosophila.. Dev Cell.

[2] Baena-López LA, Baonza A, García-Bellido A (2005). The orientation of cell divisions determines the shape of Drosophila organs.. Curr Biol.

[3] Siller KH, Doe CQ (2009). Spindle orientation during asymmetric cell division.. Nature Cell Biol.

[4] Knoblich JA (2010). Asymmetric cell division: recent developments and their implications for tumour biology.. Nat Rev Mol Cell Biol.

[5] Prehoda KE (2009). Polarization of Drosophila neuroblasts during asymmetric division.. Cold Spring Harb Perspect Biol.

[6] Chia W, Somers WG, Wang H (2008). Drosophila neuroblast asymmetric divisions: cell cycle regulators, asymmetric protein localization, and tumorigenesis.. J Cell Biol.

[7] Glotzer M (2004). Cleavage furrow positioning.. J Cell Biol.

[8] Cabernard C, Prehoda KE, Doe CQ (2010). A spindle-independent cleavage furrow positioning pathway.. Nature.

[9] Schober M, Schaefer M, Knoblich JA (1999). Bazooka recruits Inscuteable to orient asymmetric cell divisions in Drosophila neuroblasts.. Nature.

[10] Wodarz A, Ramrath A, Kuchinke U, Knust E (1999). Bazooka provides an apical cue for Inscuteable localization in Drosophila neuroblasts.. Nature.

[11] Yu F, Morin X, Cai Y, Yang X, Chia W (2000). Analysis of partner of inscuteable, a novel player of Drosophila asymmetric divisions, reveals two distinct steps in inscuteable apical localization.. Cell.

[12] Schaefer M, Shevchenko A, Shevchenko A, Knoblich JA (2000). A protein complex containing Inscuteable and the Galpha-binding protein Pins orients asymmetric cell divisions in Drosophila.. Current Biol.

[13] Kraut R, Chia W, Jan LY, Jan YN, Knoblich JA (1996). Role of inscuteable in orienting asymmetric cell divisions in Drosophila.. Nature.

[14] Siegrist SE, Doe CQ (2005). Microtubule-induced Pins/Galphai cortical polarity in Drosophila neuroblasts.. Cell.

[15] Siller KH, Cabernard C, Doe CQ (2006). The NuMA-related Mud protein binds Pins and regulates spindle orientation in Drosophila neuroblasts.. Nature Cell Biol.

[16] Siller KH, Doe CQ (2008). Lis1/dynactin regulates metaphase spindle orientation in Drosophila neuroblasts.. Dev Biol.

[17] Johnston CA, Hirono K, Prehoda KE, Doe CQ (2009). Identification of an Aurora-A/PinsLINKER/Dlg spindle orientation pathway using induced cell polarity in S2 cells.. Cell.

[18] Tio M, Zavortink M, Yang X, Chia W (1999). A functional analysis of inscuteable and its roles during Drosophila asymmetric cell divisions.. J Cell Sci.

[19] Nipper RW, Siller KH, Smith NR, Doe CQ, Prehoda KE (2007). Galphai generates multiple Pins activation states to link cortical polarity and spindle orientation in Drosophila neuroblasts.. Proc Natl Acad Sci U S A.

[20] Du Q, Macara IG (2004). Mammalian Pins is a conformational switch that links NuMA to heterotrimeric G proteins.. Cell.

[21] Moore JK, Cooper JA (2010). Coordinating mitosis with cell polarity: Molecular motors at the cell cortex.. Semin Cell Dev Biol.

[22] Lechler T, Fuchs E (2005). Asymmetric cell divisions promote stratification and differentiation of mammalian skin.. Nature.

[23] Reinsch S, Karsenti E (1994). Orientation of spindle axis and distribution of plasma membrane proteins during cell division in polarized MDCKII cells.. J Cell Biol.

[24] Gray RS, Cheung KJ, Ewald AJ (2010). Cellular mechanisms regulating epithelial morphogenesis and cancer invasion.. Curr Opin Cell Biol.

[25] Pease JC, Tirnauer JS (2011). Mitotic spindle misorientation in cancer–out of alignment and into the fire.. J Cell Sci.

[26] Egger B, Boone JQ, Stevens NR, Brand AH, Doe CQ (2007). Regulation of spindle orientation and neural stem cell fate in the Drosophila optic lobe.. Neural Dev.

[27] Poulson N, Lechler T (2010). Robust control of mitotic spindle orientation in the developing epidermis.. J Cell Biol.

[28] Zhu J, Wen W, Zheng Z, Shang Y, Wei Z (2011). LGN/mInsc and LGN/NuMA complex structures suggest distinct functions in asymmetric cell division for the Par3/mInsc/LGN and Gai/LGN/NuMA pathways.. Mol Cell.

[29] Yu F, Ong CT, Chia W, Yang X (2000). Membrane targeting and asymmetric localization of Drosophila partner of inscuteable are discrete steps controlled by distinct regions of the protein.. Mol Cell Biol.

[30] Du Q, Stukenberg PT, Macara IG (2001). A mammalian Partner of inscuteable binds NuMA and regulates mitotic spindle organization.. Nature Cell Biol.

[31] Kisurina-Evgenieva O, Mack G, Du Q, Macara I, Khodjakov A (2004). Multiple mechanisms regulate NuMA dynamics at spindle poles.. J Cell Sci.

